# Guidelines and recommendations for ensuring Good Epidemiological Practice (GEP): a guideline developed by the German Society for Epidemiology

**DOI:** 10.1007/s10654-019-00500-x

**Published:** 2019-03-04

**Authors:** Wolfgang Hoffmann, Ute Latza, Sebastian E. Baumeister, Martin Brünger, Nina Buttmann-Schweiger, Juliane Hardt, Verena Hoffmann, André Karch, Adrian Richter, Carsten Oliver Schmidt, Irene Schmidtmann, Enno Swart, Neeltje van den Berg

**Affiliations:** 1 Section Epidemiology of Health Care and Community Health, Institute for Community Medicine, Ellernholzstr. 1-2, 17489 Greifswald, Germany; 2 Unit “Prevention of Work-Related Disorders”, Division “Work and Health”, BAuA: Federal Institute for Occupational Safety and Health, Noeldnerstr. 40-42, 10317 Berlin, Germany; 3 Chair of Epidemiology, Ludwig-Maximilians-Universität München, UNIKA-T Augsburg, Augsburg, Germany; 4 Independent Research Group Clinical Epidemiology, Helmholtz Zentrum München, German Research Center for Environmental Health, Neuherberg, Germany; 5 Institute of Medical Sociology and Rehabilitation Science, Charité – Universitätsmedizin Berlin, Berlin, Germany; 6 Freie Universität Berlin, Humboldt-Universität zu Berlin, Berlin, Germany; 7 Berlin Institute of Health (BIH), Berlin, Germany; 8 Centre for Cancer Registry Data, Robert Koch-Institute, Berlin, Germany; 9 Clinical Research Unit (CRU), Berlin Institute of Health (BIH), Berlin, Germany; 10 Institute of Biometry und Clinical Epidemiology (iBikE), Charité – Universitätsmedizin Berlin, Berlin, Germany; 11 Department of Infectious Diseases and Tropical Medicine, University of Munich, Munich, Germany; 12 Helmholtz Centre for Infection Research (HZI), Brunswick, Germany; 13 Institute for Community Medicine, University Medicine Greifswald, Greifswald, Germany; 14 Institute for Medical Biometrics, Epidemiology and Informatics, (IMBEI), University Medicine, Johannes Gutenberg University, Mainz, Germany; 15 Institute for Social Medicine and Health Economics (ISMG), Otto von Guericke University Magdeburg, Magdeburg, Germany

**Keywords:** Guideline, Recommendation, Epidemiologic methods, Professional practice, Expert consensus, Research practice

## Abstract

**Objective:**

To revise the German *guidelines and recommendations for ensuring Good Epidemiological Practice* (GEP) that were developed in 1999 by the *German Society for Epidemiology* (DGEpi), evaluated and revised in 2004, supplemented in 2008, and updated in 2014.

**Methods:**

The executive board of the DGEpi tasked the third revision of the GEP. The revision was arrived as a result of a consensus-building process by a working group of the DGEpi in collaboration with other working groups of the DGEpi and with the *German Association for Medical Informatics, Biometry and Epidemiology*, the *German Society of Social Medicine and Prevention* (DGSMP), the *German Region of the International Biometric Society* (IBS-DR), the *German Technology, Methods and Infrastructure for Networked Medical Research* (TMF), and the *German Network for Health Services Research* (DNVF). The GEP also refers to related German Good Practice documents (e.g. Health Reporting, Cartographical Practice in the Healthcare System, Secondary Data Analysis).

**Results:**

The working group modified the 11 guidelines (after revision: 1 ethics, 2 research question, 3 study protocol and manual of operations, 4 data protection, 5 sample banks, 6 quality assurance, 7 data storage and documentation, 8 analysis of epidemiological data, 9 contractual framework, 10 interpretation and scientific publication, 11 communication and public health) and modified and supplemented the related recommendations. All participating scientific professional associations adopted the revised GEP.

**Conclusions:**

The revised GEP are addressed to everyone involved in the planning, preparation, execution, analysis, and evaluation of epidemiological research, as well as research institutes and funding bodies.

## Introduction

### History of the GEP development in Germany

In December 1997, an international commission on professional self regulation in science, assembled by a mandate of the executive board of the German Research Foundation (DFG), formulated the following recommendation regarding Scientific Professional societies: “Learned Societies should work out principles of good scientific practice for their area of work, make them binding for their members, and publish them.” [1] Further, the commission recommended that European professional societies pursue discussions on good scientific practice at the European level as well as nationally.

As a result of the DFG’s initiative, the *German Working Group Epidemiology* [DAE, since 2005 the *German Society for Epidemiology* (DGEpi)] authorized its Working Group on Epidemiological Methods to develop a blueprint for Guidelines and Recommendations to assure Good Epidemiological Practice (GEP). A first draft of these recommendations was openly discussed during a two-day workshop. The corrections and modifications that arose from this discussion were approved by an editorial committee and presented to the boards of the DAE and its umbrella organizations, the *German Association for Medical Informatics, the Biometry and Epidemiology* (GMDS), the *German Society of Social Medicine and Prevention* (DGSMP), and *German Region of the International Biometric Society* (IBS-DR). In an extensive consensus-building process, the recommendations were adopted by all participating professional societies in February, 2000 [2].

The GEP were evaluated based on the responses to a postal questionnaire and subsequently revised in 2004 [3]. Based on a proposal of the working group Secondary data Analysis (AGENS) of the DGSMP, the GEP was supplemented in 2008 [4] with a short update in 2014. The GEP is well accepted in Germany by a great majority of epidemiologists, public and many private sponsors as well as evaluators, peer reviewers and editors of scientific journals.

### Aim

The GEP are addressed to everyone involved in the planning, preparation, execution, analysis, and evaluation of epidemiological research, as well as research institutes, and funding bodies. The GEP adopts international best practices for epidemiological research.

The revised GEP are intended to pursue discussions of good scientific practice for epidemiological research at the national level as well as internationally. Like other internationally available good epidemiologic practice documents [5–9], they should help to eliminate scientific fraud, to ensure value and transparency in research, and to promote trusting collaborations among scientists.

The guidelines, however, should not be as limiting or inflexible as to restrain the freedom of scientific research in epidemiology. Rather, the guidelines should define the framework within which epidemiological research can be used to its fullest benefit, in all of its facets and relating to all of its areas of application.

It is quite possible that in special cases reasonable deviations from the guidelines can and sometimes even ought to be made. Such cases are capable of remaining consistent with Good Epidemiological Practice via explicit description of the nature of the deviation and its valid justification. Although many of the elements described here are already accepted as good scientific practice in epidemiology, these guidelines will be particularly important with regard to the planning and execution of future studies.

It is important for all people involved in the practice of epidemiology to be aware of the main features of good scientific practice and to implement them in daily practice. Serious cases of scientific fraud threaten the value of science itself, as they erode the public’s trust in science as well as the relationships of scientists among one another. Epidemiology, however, depends on both of these basic elements, which should therefore be safeguarded for the future by the guidelines and recommendations to assure the GEP presented here.

Comprehensiveness, usability, coverage of new developments, and acceptance of the GEP are monitored by the board of the DGEpi. Subsequent to the initial version of 1999, this has led to several new versions (see Sect. 1.1). In summer 2016 the executive board of the DGEpi decided to expand and update the GEP and tasked WH and UL with initiating a revision together with the working group on Epidemiological Methods, and the task force “Besser Forschen” (Improving Research) of the DGEpi. The main reasons for the this update were:

- After the last update in 2014, recommendations were made for specific topics within epidemiology that should be incorporated in the next revision of the GEP [10–12].
- A range of new developments and aspects has been included in epidemiologic research, e.g.:
  - new requirements on informed consent and privacy regulations
  - patient participation in research,
  - new study types and methods,
  - data sharing and open access.

## Methods

### Development of the GEP and revisions

On 31 August 2016 an inaugural meeting of the ad hoc working group took place in Munich within the framework of the annual German conference of the DGEpi. For the revision consensus was reached on a series of key points:

- recommendations made in the meantime for specific topics within epidemiology should be incorporated in the revision [10–12]. The GEP remains the common basis of all specifications. The existing ones and further specifications produced in the future supplement the guidelines of the GEP by making them more concrete and precise for specific areas of research. During the update process further guidelines, recommendations and portals, e.g. to data protection and data quality, reporting and evaluation standards, reuse of data, open access, were researched by the working group and referenced [13–19].
- In view of current developments the following new aspects, among others, were addressed:
  - explicit reference to requirements in terms of informed consent, privacy and pseudonymisation,
  - involvement of patients or study participants in the selection of research topics, questions, study designs, in particular when there are interventions,
  - new study types and methods,
  - specific quality aspects of big data analyses, and
  - data sharing and open access.

The working group edited recommendations for the revised GEP during 2017. This work formed the basis for a second consensus meeting in September 2017 at the German conference of the DGEpi. The editorial committee (WH, UL, NB) edited a draft version based on this input which was further commented upon by the working group, the executive board of the DGEpi and the spokesmen and -women of all working groups of the DGEpi. The corrections and modifications were applied by the editorial committee, and reviewed by the working group.

A second draft version with an invitation for minor comments was sent to the boards of the collaborating scientific organisations. Based on their comments, the editorial committee revised the document, and adopted the suggestions if possible. In case of plausible, however major comments that would have needed further discussion within the working group and new consensus (e.g. the comment that recommendation 3.3 lacks an explanatory text, suggestions for major cutbacks, addition of aspects of authorship in recommendation 9.1, and budget issues in guideline 3), the editing committee searched to reach an agreement with the respective contact person of the board. All participating professional associations adopted the revised GEP. This updated version was adopted in September 2018 by the board of the DGEpi. The final version is online available on the DGEpi Website since October 17, 2018 [20].

## Results: guidelines and recommendations

### Guideline 1 (ethics)

Epidemiological investigations must be conducted consistent with ethical principles and respect human dignity and human rights.

The ethical principles are derived from universal human and civil rights as well as the rights of patients, probands, and researchers [21, 22]. These ethical principles are to be applied in epidemiological research even when there is no explicit legal requirement to do so.

#### *Recommendation 1.1*

The opinion of an ethics committee should be obtained before an epidemiological study is conducted.

The basis for the evaluation is set out in the “Checkliste zur ethischen Begutachtung epidemiologischer Studien” (Checklist for the ethical evaluation of epidemiological studies) (German Working Group Epidemiology (DAE), draft 1999) [3, 23].

#### *Recommendation 1.2*

The basis of every epidemiological study is respect for the autonomy of study participants and the avoidance of unreasonable risks.

As a rule this involves documentation of informed consent.

The information provided should always contain the offer to delete the stored research data at any time, provided that they have not undergone a complete anonymisation.

#### *Recommendation 1.3*

Whenever possible, representatives of the affected populations should be involved in deciding on the research questions, defining the study endpoints and choosing the instruments.

This can occur, for instance, in the form of practice advisory boards or qualitative procedures (e.g. group discussions). In the case of suitable studies non-scientists can be involved in the selection of research topics, planning and implementation (citizen science).

### Guideline 2 (research question)

The planning of every epidemiological study requires explicit and operationalisable questions, which should be formulated specifically and precisely. The selection of the population groups to be studied must be justified in view of the research question.

The research question is the indispensable starting point for the assessment of the potential benefits of an epidemiological study. It must be apparent from the research question whether and to what extent an investigation serves medical or scientific, preventive, healthcare policy-related, socio-political or other societal interests.

The explicit formulation of the research question is an essential prerequisite for the planning and evaluation of the study design and the data collection instruments, but also the timeframe and budget of the planned investigation. It is the operationalisation of the research question which enables the development and utilisation of suitable design elements for an epidemiological study in the first place (selection of the study team, data collection instruments, estimation of the sample size to achieve the necessary precision).

The clarification and specification of the research question is a prerequisite for taking advantage of and evaluating the existing scientific evidence prior to a study. It helps to avoid obsolete hypotheses and unintended duplications of investigations.

#### *Recommendation 2.1*

In the description of the research question a distinction must be made between primary questions, for which the study was statistically optimised, and secondary questions.

#### *Recommendation 2.2*

If hypotheses are tested with a confirmatory study, these must be formulated (a priori) during the study design phase.

The use of confirmatory statistical methods requires the (a priori) formulation of the hypotheses to be tested. This must already occur in the design phase, in any case before data collection begins. The basis for these hypotheses is a research question which is operationalisable, quantifiable and testable.

#### *Recommendation 2.3*

If data analysis goes beyond the original, primary purpose (secondary data [10]) or the primary reason for data collection, this is to be indicated in the methodology.

Transparency about the primary purpose, the data collection rules and analysis procedures must be created. When pre-existing data are used the original collection context and purpose must always be named and taken into account. The restrictions which result from the characteristics of secondary data are to be taken into consideration in the interpretation of the results.

#### *Recommendation 2.4*

The use of research methods without pre-defined hypotheses can be justified.

The availability of *big data*, including mass data (e.g. *OMICS data, sensor data, image data*) can limit the use of conventional statistical methods [24, 25]. In this case it can be justified to make use of modern data science methods that do not pre-specify hypotheses. The limitations of the study design, the data source and the analysis method must be named when presenting the results.

A lack of coding standards or an unstructured and/or incomplete form of data collection can potentially distort the results. With regard to the method applied, it must be stated how, for example, confounding by indication, sampling bias, selection bias, ascertainment bias and overestimation of effects are handled and how the results can be interpreted in comparison with conventional statistical methods [26, 27]. An example is results from machine learning algorithms, for which a validation using external data is recommended.

### Guideline 3 (study protocol and manual of operations)

The basis of an epidemiological study is a detailed and binding study protocol, in which the study characteristics are set out in writing.

The creation of a study protocol/study plan before the commencement of a study is a fundamental methodological requirement for the quality of the study. The study protocol is a compilation of the most important information necessary to apply for grants and for assessment of a study as a research project and its conduct.

The following should be components of the study protocol:

- research question, working hypotheses where applicable
- study type
- target population, source population and study population
- scope of the study (number of subjects, in the case of cohort studies also the observation period) and the rationale behind it
- procedure for the selection and recruitment of the study participants
- definition of and measurement and data collection procedures for
  - the target variables (endpoints)
  - exposures or risk factors
  - potential confounders, effect modifiers and mediators
  - stratification variables (e.g. for subgroup analyses)
- measures to reduce bias
- naming of all data sources
- concept for data collection and storage
- data management
- analysis strategy including the statistical models
- quality assurance measures
- handling of clinical/incidental findings
- measures to ensure data protection and adherence to ethical principles
- timetable with a definition of the responsibilities.
- the responsibilities for and process of issuing amendments as well as their evaluation, enactment, and implementation.^1^

Existing recommendations for the design and analysis of observational studies [28] and aspects of study and reporting quality in accordance with the respective current reporting guidelines (cf. Recommendation 10.1) should be taken into account when the study is being planned [10, 11, 29].

#### *Recommendation 3.1*

The study type should be described and its selection adequately justified.

The choice of the study design depends on the methodological considerations which arise from the research question, the freqency of the diseases in question and the influencing factors which are of interest, the scaling of the target variables, strategies to avoid bias, and the available resources (accessibility of data sources, study participants and cohorts, expenditure, duration). Published studies with a comparable objective are also important.

Epidemiological study types encompass all study designs which are suitable for the purpose of answering epidemiological research questions. The terminology is diverse and not always uniform. These include, alongside the classical observational studies (cohort, case–control and cross-sectional studies), diagnostic studies, other descriptive representations of prevalence or incidence (e.g. surveillance or register studies, monitoring), ecological studies (correlation studies or studies with aggregated data) as well as mixed or modern study designs (e.g. case-cohort, case-crossover or case-only studies). Epidemiological intervention studies, by contrast with clinical studies, are oriented more towards primarily healthy populations in various settings or groups. Apart from experimental studies with randomisation (e.g. cluster randomised studies), interventions without random allocation (e.g. pragmatic studies or before/after studies) are possible. It is possible to complement epidemiological studies by means of a combination with qualitative methods (mixed methods approach). Various forms of reviews and meta-analyses which include epidemiological primary studies, add, as secondary research, to the spectrum of epidemiological study types [30–32].

#### *Recommendation 3.2*

The source population and the procedure for selection of the study participants should be described and adequately justified.

Both, the external validity (generalisability) and in certain cases also the internal validity of the study results depend to a great extent on the choice of the source population and the procedure used to select the study participants. Differences in the incidence of the diseases to be investigated or influencing factors such as the availability or the comparability of the information gathered often make it necessary to limit the source population to particular subpopulations. Both inclusion and exclusion criteria should be defined a priori and adequately justified. For example, the study design and methodology should ensure that the gender- and age specific aspects can be adequately adressed. In the case of topics and questions which pertain to all genders and/or all age groups, a justification is required if only a specific gender and/or age group is included.

#### *Recommendation 3.3*

The selection of the study participants must ensure that the prerequisites for the statistical method to be used are met.

#### *Recommendation 3.4*

In the planning stage of epidemiological studies possible distortions of the results (bias) should be counteracted.

When planning a study, measures should already be taken to prevent bias, which can arise through selection, measurement errors and confounding etc. These measures can include, for example, a reduction in the variability of confounders through a limitation of the random sample selection or the collection of information, which is necessary, for instance, for the control of confounding factors. In order to estimate the effect of bias on the results of the study additional data collections should be planned, for example to check the validity of measurement procedures, as well as validation and calibration. Sensitivity analyses should be conducted to examine possible bias.

#### *Recommendation 3.5*

The concept for minimisation and control of potential selection bias due to non-response should be documented in the study protocol.

Such a concept includes participant-related documentation of the reasons for non-participation or the later exclusion from the study. In the course of the study an attempt should be made to also obtain basic information from the non-participants. The goal of the collection is to predict the direction and extent of a possible non-response bias. For the documentation the various categories of non-participation must be defined in advance and continually recorded. For a detailed response analysis both successful and unsuccessful contact attempts should be documented according to type, content and timing.

In order to evaluate possible non-response bias and compare studies, at least the following categories should be included in the report of the results of an epidemiological study:

The number of subjects:

- who provided complete data
- who provided partially complete data
- who refused to participate
- who were too sick to take part
- who were not eligible according to the study protocol, in other words who did not fulfil the inclusion criteria and/or met the exclusion criteria
- who could not be reached (those who moved to a new address and the deceased are to be recorded separately).

To avoid selection effects a stratified analysis of the response is necessary, for example according to gender and age. For cohort studies the reasons for the premature withdrawal from the study are to be recorded and reported.

#### *Recommendation 3.6*

All variables of interest should be precisely defined and operationalised according to existing standards whenever possible. Measurement and data collection instruments which are as valid and reliable as possible are to be employed. If they exist standard terms should be used [33].

In addition to the qualitative description, information should also be provided, in particular for exposures, about the quantity and the timing. Diseases or causes of death should be defined and coded using internationally recognised diagnostic standards. In addition, for classifications of diagnoses, medical services, degrees of severity etc. internationally recognised codes should be used (e.g. ICD, TNM, NYHA classification etc.).

The measurement properties, including validity and reliability, of the instruments used should be presented (e.g. according to sex, age). Where possible, standardised and validated instruments are to be used [34]. The choice of the measurement and data collection instruments used should be justified.

#### *Recommendation 3.7*

All the data sources used are to be named in the study protocol.

All the data sources from which information on the study populations is obtained should always be described (hospital discharge diagnoses, death certificates, surveys of the subjects, workplace accounts from occupational medicine sites, geographical data etc.).

#### *Recommendation 3.8*

In the study protocol a justification and quantitative assessment of the appropriate sample size is to be given.

The assessment of the scope of the study serves not only to put a figure on the estimated expenditure of resources (costs, working hours etc.) for the answering of the epidemiological question. Beyond that, it should also be shown that an appropriate, and in a certain sense also optimal, relationship exists between the resource cost and benefit (in terms of the anticipated accuracy of the results using the chosen method of statistical analysis).

The assumptions which are the basis for this assessment, for instance with regard to the expected effect size, the prevalence and incidence of the endpoints of interest, the level of significance and statistical power, the value above which an effect size is relevant etc., should be explicitly stated.

#### *Recommendation 3.9*

For the analysis phase of the study sufficient time and human and technical resources must be included in the study protocol.

Proper analysis of the data in epidemiological studies is only possible if sufficient time, suitably qualified staff and technical equipment is available [35].

#### *Recommendation 3.10*

In addition to the study protocol all organisational stipulations with regard to the preparation and implementation of the study, including the data collection instruments, should be documented in a manual of operations.

For epidemiological studies a manual of operations should be drawn up with all the standard operating procedures (SOPs) specified for the study. Here, in addition to the data collection instruments used, organisational requirements with regard to the timetable and procedure, allocation of staff, methods to be used for contacting and recruiting the study participants, technical procedures (e.g. laboratory tests) should be formulated in advance. Furthermore, the preparatory steps like interviewer training, the organisational measures for quality assurance and quality control, and the ongoing evaluation during the process should also be described in SOPs.

#### *Recommendation 3.11*

The study design and study protocol should be registered and where possible published.

The study design and protocol should preferably be disclosed prior to the commencement of the study in order to increase the transparency of the research project and to reduce the publication bias.

### Guideline 4 (data protection)

When planning and conducting epidemiological studies attention should be paid to ensuring compliance with the applicable data protection rules and the safeguarding of informational self-determination.

All persons who handle personal data within the framework of a research project must be informed about the contents and reach of the relevant legal provisions as well as the associated possibilities. In research with personal data it is necessary to take into account the right of the individual to informational self-determination, but also the right to freedom of science and research and the knowledge gained, which benefits society at large. Those active in epidemiology should consequently and actively emphasize the interests of the research community and work towards improvements in the data protection regulations with regard to the use of personal data for scientific purposes.

The storage, evaluation, transmission and publication of anonymised data are not subject to any restrictions associated with data protection except that they are to exclusively be used for scientific research and possibly the obligation to delete the data once the goal of the research has been achieved [36, 37].

The EU General Data Protection Regulation (2016/679) defines personal data as any information, which is related to an identified or an identifiable natural person. As a rule, these are to be pseudonymised in epidemiological studies [38].

#### *Recommendation 4.1*

All persons who handle personal data within the framework of a research project must be informed about the contents and reach of the relevant legal provisions and the associated possibilities and confirm this in writing.

#### *Recommendation 4.2*

Personally identifying data and research data must be stored separately.

The protection of personally identifying data (name, birthday, place of residence) is of particular importance. This can be achieved for example by implementing independent networks or network zones as well as by the strict division of people with access rights to personally identifying data and those with access to research data. In the case of suitable studies an independent trust centre for personally identifying data can be employed [39].

#### *Recommendation 4.3*

Efforts should be made to achieve a differentiated management of patient identifiers (PID).

For the data collection phase and also the later use of the data, usage of different PIDs is recommended. In the case of complex studies making use of data sources from various organisations the usage of source-specific PIDs is still advisable.

The use of different PIDs makes it easier to maintain high data protection standards because it becomes more difficult to unintentionally link separate datasets.

### Guideline 5 (sample banks)

In many epidemiological studies it is necessary or useful to set up a biological sample bank. For the current and the intended future use of the samples it is a requirement that the consent of all subjects is documented.

In many epidemiological studies it is necessary or useful to set up banks of biological samples (e.g. serum, whole blood, other bodily fluids and biological tissue). Even when analyses of the samples are carried out immediately during the course of the study, a simultaneous analysis of all samples upon completion of subject recruitment is often necessary in order to ensure uniform laboratory procedures while maintaining the highest possible quality standards. Since the recruitment of the test subjects takes place over a longer period of time in most epidemiological studies, the collection of biological samples thus almost always involves setting up a sample bank, at least for the duration of the study.

Furthermore, in many cases it makes sense to store biological samples in sample banks beyond the duration of the study. This makes it possible, among other things, to test the reproducibility of the results if there are doubts as to the validity of the laboratory analyses. This also enables more reliable or more differentiated analyses of the primary research questions of the study to be conducted later using laboratory techniques which have been further developed and improved in the meantime, and also enables the analysis of additional markers that have since been identified, which can be of significance as potential risk factors in their own right and also as potential effect modifiers, effect mediators or confounders. The necessity to safeguard the long-term preservation and accessibility of biological samples and the possibility of later testing arises in particular for prospective long-term cohort studies, the evaluation of which occurs, in many cases, decades after the collection of the biological samples.

At the same time, it must be ensured that the subjects are fully informed about the storage and the current and planned future use of the biological samples, and that procedures are established for the deletion or destruction of an individual biological sample. The procedures for a possible communication of the results of laboratory analyses to the subjects, as well as for guaranteeing the confidentiality of the results, are to be clearly regulated. This concerns in particular the determination of parameters with high individual significance for disease risks, diagnosis, prophylaxis and treatment, e.g. certain genetic analyses [39, 40].

#### *Recommendation 5.1*

The institution and people who are responsible for running the sample bank should be made known to the study participants. At the same time, the type and quantity of the biological material collected should be described along with the storage method, location and duration. The subjects are to be informed about the ownership status of the material taken.

Conflicts of interest, such as within the framework of commercial co-operations, must be stated. The informed consent should always contain the offer to terminate the storage of material in the sample bank at any time, as long as complete anonymisation has not taken place.

#### *Recommendation 5.2*

When material preserved in sample banks is used for research questions which were not originally planned, the GEP is again to be observed.

Before conducting later investigations, which were not yet foreseen at the time informed consent was obtained from the subjects (e.g. data pooling, merging of samples within the framework of international studies), the requirements, such as, for instance, once again obtaining a declaration of consent, the degree of anonymisation, communication of the results to the subjects etc. are to be assessed separately with renewed involvement of a relevant ethics committee.

### Guideline 6 (quality assurance)

In epidemiological studies, an accompanying quality assurance of all relevant instruments and procedures is to be guaranteed.

Internal quality assurance is an essential component of every epidemiological study. It is to be safeguarded through the description of its contents and the naming of those responsible. Due to the associated costs its scope must be in an appropriate relationship to the overall scope and costs of the study. The target of the quality assurance is the timeframe and organisational and technical implementation rules set out in the study protocol and manual of operations.

#### *Recommendation 6.1*

In every epidemiological investigation in which primary data are collected it must be determined whether a separate pilot study is necessary before the start of the main study.

Pilot study in the narrower sense is here understood to mean a simulation of the main study or the testing of essential elements of the main study. A pilot study thereby differs from a pilot phase (run-in phase). Procedures and process sequences including data collection methods are tested and used in an identical way to in the planned main study, just on a smaller scale. A pilot study will, as a general rule, be necessary if an unusual sample is drawn, if there are unfamiliar contact requirements or if other aspects relevant to the study are still untested. Where required, a validation of instruments can also take place in the context of a pilot study prior to planned research.

The pilot study should be evaluated and documented before the start of the main study so that any modifications which may be required can be inserted into the study protocol and manual of operations of the main study.

#### *Recommendation 6.2*

Modifications to the instruments in a study or the introduction of new instruments are to be checked using pretests.

A pretest describes, in contrast to a pilot study, the targeted simulation of an examination or a group of examinations. In contrast to a pilot study the procedure for the entire study is not simulated. A pretest will generally be necessary when a new data collection instrument or medium or a new access method is to be used. If necessary, a validation of instruments can also occur within the scope of a pretest for a planned study. All data protection and ethical aspects are to be taken into account even in pilot studies and pretests.

#### *Recommendation 6.3*

If a change to the established procedures (amendment) becomes necessary during the study, then these changes are to be justified, to be documented in writing and to be announced to all study personnel in time.

An amendment should be submitted to the competent ethics committee.

#### *Recommendation 6.4*

Before the start of fieldwork the people involved in the data collection should be thoroughly prepared and trained and their activities should be constantly monitored.

The data collection personnel are to be carefully selected and it must be ensured that they are qualified, both socially and professionally. In the course of the collection, where appropriate, the training should be refreshed. In larger studies with a long duration and/or multi-centre studies there should be regular checks in a structured manner (certification, recertification) to guarantee the activities are carried out correctly.

#### *Recommendation 6.5*

Especially in large, long-lasting and multi-centre investigations, it should be determined whether quality assurance of the processes should be carried out by an external individual or institution.

External quality assurance does not replace the internal quality assurance, but rather it scrutinises its processes, results and consequences. When applying for funding, where appropriate, funds for external quality assurance are to be taken into account.

#### *Recommendation 6.6*

Data collections should be accompanied by a continuous data monitoring with standardised reporting.

In order to discover irregularities in the data at an early stage, epidemiological data collections should be accompanied by standardised data monitoring. This should focus on aspects like missing values, implausible values or value distributions.

#### *Recommendation 6.7*

Data collection and quality assurance should not be performed by the same person or group of people.

Efficient quality management is a highly complex task, which, whenever possible, should be entrusted to a team or individuals who are specially trained. If possible, the quality assurance role should be filled from outside the investigative team, in order to avoid conflicts of interest.

#### *Recommendation 6.8*

The data collection should be carried out electronically whenever possible.

The collection method should be adapted to the population and situation and be justified. If the type of data allows it, manual data entries should preferably be avoided. Instead, in all cases where it is possible, electronic recording of data using eCRFs (electronic Case Report Forms) is to be preferred [41].

### Guideline 7 (data storage and documentation)

A detailed concept is to be developed in advance for the recording and storage of all the data collected during the study, as well as for the processing, plausibility checks, coding and provision of the data.

#### *Recommendation 7.1*

For the recording and storage of the data, uniform data management should be employed.

For logistical and quality reasons, centralised storage of the data is preferable. In the case of a decentralised approach it is necessary to pay attention to the maintenance of uniform standards.

Given the complexity of efficient and high quality data management a manual should be produced for this purpose.

#### *Recommendation 7.2*

Before the commencement of data collection, a complete data dictionary is to be drawn up and, if necessary, adapted during the course of the study.

The data dictionary contains all the necessary information for data recording for all the planned measurement instruments (e.g. variable names, labels, response categories, missing value codes, possibly other metadata), logical rules for modelling the dependencies of variables and investigations as well as information relevant for quality assurance such as plausibility limits for numeric variables.

#### *Recommendation 7.3*

All data collected during the study (documentation forms, questionnaires, measured values and laboratory values etc.) should be promptly transferred to a database, which ensures reliable and secure recording and storage of the data.

A database structure is a prerequisite for regular data checks, which must be carried out in a timely manner in parallel to the field work. In this way qualitative and quantitative deficiencies in the data can already be identified during the fieldwork phase and appropriate interventions can take place. The original documents should be kept in an appropriate form (originals, scans, raw datasets or similar) until at least 10 years after the end of the study.

The recording of plaintexts enables later testing of the assigned codes and additionally makes the plaintexts available for later more in-depth analyses and is thus expressly recommended.

Whenever data which contains manual elements is recorded, a second entry should be made, where possible by a different person. For the second entry particular attention must be paid to those variables to which there would only be limited access for a later plausibility check (e.g. age, date, calendar year), however, if possible it should include all the numeric variables.

When data is recorded electronically the possibility of implementing automatic test algorithms should be examined.

#### *Recommendation 7.4*

The raw dataset obtained after the test entry should be stored in an unchanged form.

The raw dataset should generally be kept in an appropriate form for a period of at least 10 years after the conclusion of the study. It serves, for instance, as a starting point for subsequent validation work. Complete transparency with regard to the data preparation and evaluation steps, for example in the context of external quality assurance, can only be guaranteed with the raw dataset.

#### *Recommendation 7.5*

Coding of data must always occur independently, i.e., blind to the status or group membership of the person in question. Standard classifications should be used, if available.

In many cases a categorisation with subsequent coding is necessary. Every coding of plaintexts should be carried out on the basis of standard classifications (e.g. ICD-classification, occupational classification, industrial classification). As a quality assurance measure an independent second coding is recommended. Where this is not possible, a check of the quality of the coding can be carried out using a random sample, it should however, when the results warrant it, be extended to cover the entire dataset.

During the coding of data an effort must be made to ensure a blinding which is as extensive as possible with regard to the case status and exposure status.

#### *Recommendation 7.6*

Plausibility checks take place, in principle, on the basis of the verified raw dataset. Changes to the variable values, if necessary, or the creation of new variables should be carried out by means of a script wherever possible and are to be documented in writing.

Some of the plausibility checks can already take place during data entry with an appropriate mask control, whereby in particular permissible value ranges and compliance with the filtering procedure are to be checked or safeguarded. In individual cases original data collection documents or other raw data sources (e.g. audio recordings) can be referred to in order to check implausible entries.

The documentation of changes to the variable values should contain at least the following information:

- date of the change
- variable name
- old variable value
- new variable value
- type of error/reason for the change
- person carrying it out

#### *Recommendation 7.7*

The revised dataset after plausibility checks and correction of the data is to be labelled the analysis dataset and is to be saved separately from the raw dataset.

The creation of revised analysis datasets, which may in some circumstances be required after data revisions or plausibility checks, must be clearly documented and the programme script is to be kept. Recourse to the raw data for a later review of the results must be possible at any time (regarding the duration see 7.4).

#### *Recommendation 7.8*

Research data should—taking into account data protection law, ethics and other relevant aspects—be made available for reuse.

Here, the so-called FAIR-principles are to be observed [11]:

1. the data should be able to be found (findable), among other things through the use of public repositories [Answers to frequently asked questions about this topic are compiled in the “Handreichung Open Access und Repositorien” (Open Access and Repositories Handout)], through identifiability by means of a persistent identifier (e.g. DOI) and the persistence of the data storage over a long, at least 10-year period,
2. the access to data should, in the case of research questions which are in the public interest, be as extensive as possible (accessible), access restrictions may however be appropriate e.g. by means of Scientific Use Files among other things for data protection reasons (potentially the establishment of a data trust),
3. the data format chosen should be compatible with conventional data formats (interoperable) and
4. the data should be able to be reused (re-usable), i.e. the data should, among other things through the provision of metadata, be as simple as possible to use (inter alia code plan, data dictionary).

Access by third parties should as a rule be formalised by means of a transparent use & access regulation and clearly defined conditions of use. In the case of particular data (e.g. secondary data, especially social data, geographical data) there may be special features which need to be taken into account [10, 11].

There is often more information available in collected data than those responsible for the study can use themselves. Data and scripts released as the result of research activities are also publishable and can be cited and increase the visibility and recognition of the data producers. It is therefore recommended to allow other scientific institutions to make use of the data, where appropriate with a contractual arrangement [42]. The necessary time, human and technical resources/infrastructure should already be planned and budgeted for at the beginning of the study and the required resources should be weighed up against the reuse potential.

#### *Recommendation 7.9*

If data collections are made available for reuse, rules of procedure to protect the personal rights of the subjects as well as to avoid misuse of the data must be adequately implemented.

Not all research data in epidemiology can and should be made universally freely available. The size of the data collections and the, in some instances, high proportion of those examined in terms of the total population involve the risk of re-identification of the subjects and the misuse of the data. On the other hand, epidemiological data are often financed by taxpayers and thus public in nature [19, 43]. For this reason, before the provision of the data occurs, careful consideration must be given to how access to the data can be arranged. A data management concept created at the start of the study can already be helpful early on in creating a shared understanding with the various project participants of the work with the data, which data can be made available for reuse, which additional documents need to be prepared for this purpose (e.g. study protocol, analysis plan, etc.), when the data should be made available and what form of access would be possible for its reuse.

In order to ensure that those responsible for the study can be reliably identified and also remain referenceable, an obvious choice is the use of a unique ID for scientists and academics (e.g. Open Researcher and Contributor ID: ORCID).

### Guideline 8 (analysis of epidemiological data)

The analysis of epidemiological data should be performed using adequate methods and without undue delay. The data underlying the results are to be stored in a fully reproducible form for at least 10 years after the end of the study.

The analysis of epidemiological studies should be valid, transparent and take place promptly on the basis of the specifications set out in the analysis concept in the study protocol, and should be comprehensible for third parties at all times. The call for a prompt analysis of epidemiological studies generally arises as a result of the public interest in the results.

Investigations for instance of risks in the workplace or in relation to environmental pollution are often health policy-driven and conducted on behalf of authorities, ministries inter alia. These clients have the right to the earliest possible completion of the most important analyses to enable them to effectively fulfil their mandate of averting damage to the health of the population.

#### *Recommendation 8.1*

The analysis of the individual research questions should occur according to an analysis plan created beforehand.

The analysis plan contains the specifications of the data and variables to be included alongside the procedure for the selection and adaptation of models and the statistical methods to be used, the handling of implausible or missing values, outliers and sensitivity analyses.

The sophistication of the analysis plan must be proportionate to the objective of the study and the prior knowledge.

#### *Recommendation 8.2*

Prior to working on the scientific questions the scientists should become thoroughly familiar with the collection context and the characteristics of the data.

Before the start of the actual scientific analyses it is necessary for the scientists to have detailed knowledge of the data and their characteristics so that they can avoid analysis errors or so that they can conduct analyses optimally. The results of this initial step are to be documented.

#### *Recommendation 8.3*

Evaluations before the end of data collection should only be carried out when justified.

Epidemiological studies should not, as a rule, with the exception of longitudinal studies, be evaluated until after completion of the recruitment and data collection. If interim analyses are planned these should be mentioned and justified in the study protocol. Unplanned interim analyses can seem to make sense in exceptional cases because of urgent research questions, however, they are then to be explicitly justified before the beginning of the analysis. Evaluations before data collection has been completed can however be useful in particular when dealing with methodological issues.

#### *Recommendation 8.4*

Analyses of epidemiological study data should be peer reviewed before publication. The underlying data and programmes should then subsequently be archived in a fully reproducible form.

Data should be provided to give the co-authors the possibility to reproduce parts of the analysis themselves. In order to prevent incorrect analyses finding their way into a publication, it is advisable to have all the results reproduced by a suitable person, who had not previously been involved in the evaluations. Inconsistencies in the results between the original analysis and the independent peer review require complete clarification; consistency on the other hand proves the reproducibility of the results on the basis of the described procedure.

Before the peer reviewed analyses are published as scientific results (talks at national or international conferences, publicly available reports, original work in scientific journals), it must be ensured that the evaluation strategy, the analyses and their results are reproducible by third parties. Secure archiving of all datasets relevant to publication and the associated evaluation scripts is necessary.

In addition, there is an obligation to unambiguously indicate the analysis dataset used for the analysis, with the name, creation date and also storage location. This also includes a transparent documentation of all new variables created in the analysis process (transformations, links etc.) as well as all evaluation scripts.

All analyses should be documented in such a way that external people or institutions can understand and reproduce the analysis strategy, the actual analyses and their results.

#### *Recommendation 8.5*

Statistical code should be used wherever possible.

Analyses must be completely documented to ensure transparency and comprehensibility, e.g. in the form of the evaluation scripts used. If graphic user interfaces (e. g. menus) are used, all programme steps must be documented in a suitable form [44].

#### *Recommendation 8.6*

When re-using others’ data for one’s own research there is an obligation to adequately cite the underlying data.

Quality-assured research data are a cornerstone of scientific knowledge and, independent of the purpose for which they were originally obtained, can often serve as the basis for further research. The following principles should be considered when citing data [45, 46]:

1. Data should be seen as legitimate products of research which can be cited (importance). Data citations hereby attain the same status as other research results, for example publications.
2. Through the citation of data the production of research data is recognised as a primary scientific achievement (credit and attribution).
3. In scientific debate the underlying datasets should be cited if they are used as the basis for argumentation (evidence) [47].

### Guideline 9 (contractual framework)

To carry out an epidemiological study a defined legal and financial framework is required. To this end, legally binding agreements should be sought between those providing funding and those being funded as well as between research cooperation partners.

Larger epidemiological studies are today generally externally funded, at least to a significant extent. The providers of funding can be research promoting institutions or equally clients from the public or private sector. The charters of some research institutes dictate the parameters for conducting externally funded research. Many funding providers also have specifications, conditions and restrictions to take into account when commissioning research.

#### *Recommendation 9.1*

Written agreements should always be made with all cooperation partners. This applies regardless of whether they are study centres on an equal footing within the scope of a multi-centre study or if it is a cooperation partner working on one or several work packages as a contractor within a larger study project.

In the agreement the following points should be specified:

- Structure and allocation of tasks within the research project
- Overall timetable of the proposed research project and the timetables of all cooperation partners
- Overall financing plan and allocation of funds
- Recommendation regarding compliance with the GEP guidelines
- Obligatory measures for quality control and assurance
- Instruments and procedures used
- Procedure and conditions for awarding subcontracts to third parties
- External presentation, media and public relations
- Access and utilisation rights to the jointly collected data during the data acquisition and after completion of the research project
- Publication agreement
- Plans for the long-term storage of the raw data
- Procedure for analyses which go beyond the primary and secondary hypotheses of the research project or the subject matter of the contract
- Procedure in cases of dispute
- Conditions, rights and procedure for the termination of the contract, the scope and form of handover of contractual obligations which were partially fulfilled prior to the termination
- Procedure if a study is discontinued

#### *Recommendation 9.2*

Transparent and realistic agreements which preserve the independence of the research must be made with the sponsors, clients and cooperation partners. Due to the numerous potential constellations, various forms of contract are possible.

The following aspects are to be taken into account:

- *Independence of research* An ongoing research project cannot be terminated before its completion by the sponsor, client or cooperation partners without there being serious objective reasons for doing so. The responsibility for compliance with GEP lies exclusively with the lead investigator(s), or rather, with the scientists who have been entrusted with the task.
- *Supervision and monitoring* The type and scope of external supervision, control and testing procedures by the sponsors and clients should be specified in the agreement.
- *Long*-*term access to the data* The lead investigator(s) and/or client must ensure that the dataset underlying a publication remains available for at least 10 years after publication. Additionally, for further analyses by external data users, the duration, extent and conditions must be regulated in contractual agreements (change of institution, legal succession, secondary analyses etc.).

#### *Recommendation 9.3*

The publication of the results is not allowed to be prevented, obstructed or unreasonably delayed.

Non-disclosure periods and the participation rights of the sponsor, the client or cooperation partners must be explicitly stated in the contracts and the agreements and the scope must be specified and justified. For projects that receive public funding, it must be ensured that public scientific discussion and scientific publication cannot be denied to the contractor by the client, for example through the sponsors, beyond reasonable non-disclosure periods. For scientists from research institutions this also applies by analogy for their own institution. The production of the publication is generally the responsibility of the head of the study. In other cases the lead investigators are to be granted unrestricted participatory rights.

#### *Recommendation 9.4*

The legal framework for the use of routine data is to be adequately taken into account.

Epidemiological research increasingly makes use of social data and health-care data from clinical routine. The successful implementation of such projects is dependent on the conditions of the relevant legal framework being given appropriate consideration, e.g. federal and state data protection laws, social legislation [47].

### Guideline 10 (interpretation and scientific publication)

The interpretation of the research results of an epidemiological study is based on a critical discussion of the methods, data and results in the context of the existing evidence. The interpretation is the responsibility of the scientists. All publications should undergo an external review.

Alongside personal integrity and objectivity, technical and methodological professionalism, comprehensive information and adherence to scientific criteria are necessary prerequisites for a proper interpretation of epidemiological study results. The assessment of the results must therefore not be left solely to the clients, political decision-makers or the media. Rather, it is one of the core tasks of the responsible scientific head of a research project and the authors of the respective publication. The epidemiological expert must present the argumentation underlying the interpretation in a transparent and comprehensible way in a written discussion.

As a general rule research results should be subjected to an independent review by experts (peer review). In contrast to the internal checks to verify the reproducibility of the analyses, with external reviews the emphasis is placed on the validity of the study design, the analysis strategy and the interpretation.

#### *Recommendation 10.1*

In reports and publications of epidemiological studies the relevant reporting standards should be used [48–55].

An overview and collection of all current, relevant reporting guidelines is offered by the EQUATOR network (Enhancing the QUAlity and Transparency Of health Research) [52]. Selected relevant recommendations for epidemiological studies are the STROBE Statement [53], the STROBE-ME Statement [54], the RECORD Statement [50], STROSA [57] and the PRISMA Statement [51]. Further guidelines and recommendations are offered by Technology, Methods and Infrastructure for Networked Medical Research (TMF).

#### *Recommendation 10.2*

The goal should be for scientific publication to occur in open access formats.

Open access (OA) describes unrestricted access to scientific information free of charge. It is also possible to promptly make articles published in conventional journals publicly available (i.e. at the time of publication or shortly thereafter) in repositories or document servers [56].

### Guideline 11 (communication and public health)

Epidemiological studies should appropriately involve the affected population groups.

Epidemiologists should convey the results of their studies to the affected population groups and other actors (e.g. social partners, politicians, self-governance organisations) in easy-to-understand language and choose suitable means of access and formats to do so. The professional communication with interested members of the public is also the task of the epidemiologist.

#### *Recommendation 11.1*

If, in the professional opinion of the responsible epidemiologists, the research results of an epidemiological study indicate the need for consequences, these should be explicitly formulated, for instance in the form of a recommendation. Epidemiologists are also responsible for the risk communication with non-epidemiologists where required [57].

The results of epidemiological studies and the risk assessments derived from them give rise to misinterpretations in the media time and time again, but also among interested members of the public and politicians. In some instances this brings epidemiology itself into disrepute as a science. Epidemiologists should generally take part in the discussion and enable objective risk communication through high-profile contributions. In this way they can contribute to the informed handling of the respective study results and the uncertainties possibly associated with them.

#### *Recommendation 11.2*

The instruments and procedures used in a study should be disclosed to interested parties.

This is both a confidence-building and at the same time a quality assurance measure in the interests of the transparency of epidemiological results and is a safeguard against accusations of result manipulation.

## Concluding remarks and future directions

GEPs are meant to be regularly revised [7]. The goal of this revision was to update and further develop the GEP including new thematic areas, e.g. research questions and methods, requirements concerning informed consent, privacy regulations, patient participation, and research involving “big data” analyses. The revised GEP consists of 11 guidelines (1 ethics, 2 research question, 3 study protocol and manual of operations, 4 data protection, 5 sample banks, 6 quality assurance, 7 data storage and documentation, 8 analysis of epidemiological data, 9 contractual framework, 10 interpretation and scientific publication, 11 communication and public health) and modified and supplemented the pertinent recommendations to each of the guidelines. The revised GEP also comprises related German Good Practice documents (Secondary Data Analysis, Health Reporting, Cartographical Practice in the Healthcare System).Some aspects of the GEP are specific for the German situation, e.g. a conflict of the research community with regard to data protection regulations in guideline 4 (data protection), that hinder for epidemiologic research. Further aspects may be derived from the comments from the other scientific associations (GMDS, the DGSMP, and the IBS-DR) that could not be incorporated due to time constraints, and concurrently developed GEP documents from other countries [8, 9]. Valuable input for a next revision was also given by the reviewers of this article. Particularly some recommendations were considered to be too restrictive. Language changes (“may instead of “must) were suggested for recommendations 2.1 to 2.3, a less rigid concept of “apriori” in recommendations 3.2 and 8.1, and longer data storage in recommendation 3.8. Further development may include a stronger focus on relevance to the society that might be adopted from the recent GEP guideline from the Netherlands Epidemiological Society [8]. The still preliminary guidelines for GEP in global health research developed by the KIT Royal Tropical Institute staff [9] that were following the AGREE II methodology may provide valuable input for the process of guideline development in the field of epidemiologic methods. The KIT GEP are currently being piloted internally before finalisation. Further developments may also apply guideline development manuals (such as the German Network of Scientific Medical Societies in Germany [58]). This may include externally moderated formalized consensus procedures and systematic literature search for evidence where applicable (e.g. increased reliability by a second entry of data in recommendation 7.3). It has to be kept in mind, that this requires additional time and funding. A cheaper possibility would be to evaluate the German GEP with the AGREEII and make adjustments were needed. Within an appropriate time period, e.g. after 2 years, a conference of the DGEpi can be organized with interested members from the collaborating associations DGSMP, GMDS, DR-IBS, TMF, DNVF and other related societies in which the usability of the GEP and new aspects are discussed in order to prepare a revision in 2023. This may also include other medical scientific societies with a methodological binding to epidemiological research (like occupational and environmental medicine).

## Footnotes

1 This point was added as a consequence of the peer-reviewing process of the English version of the GEP.

## References

[1] Proposals for safeguarding good scientific practice. Recommendations of the commission on professional self regulation in science. Memorandum. Deutsche Forschungsgemeinschaft. http://www.dfg.de/download/pdf/dfg_im_profil/reden_stellungnahmen/download/empfehlung_wiss_praxis_1310.pdf (1997). Accessed 19 Oct 2018.

[2] Leitlinien und Empfehlungen zur Sicherung von Guter Epidemiologischer Praxis (GEP) [Guidelines and recommendations for insuring good pidemiological ractice (GEP). German Professional Society of Epidemiology]. Gesundheitswesen 2000;62:295–303. Version 1999 can be retrieved from http://www.rki.de/DE/Content/Gesundheitsmonitoring/Studien/Methodik/Empfehlungen/empfehlungen_pdf1.pdf. Accessed 23 Oct 2018. 10893878

[3] Hoffmann W, Latza U (2005). Terschüren C [Guidelines and recommendations for ensuring Good Epidemiological Practice (GEP)—revised version after evaluation]. Gesundheitswesen.

[4] Guidelines and recommendations for ensuring Good Epidemiological Practice (GEP). German Society for Epidemiology (DGEpi). https://www.dgepi.de/assets/Leitlinien-und-Empfehlungen/cec55ccaaa/Recommendations-for-good-Epidemiologic-Practice.pdf (2018). Accessed 23 Oct 2018. 10.1007/s10654-019-00500-x PMC6447506 30830562

[5] Guidelines for good epidemiology practices for occupational and environmental epidemiologic research. The Chemical Manufacturers Association’s Epidemiology Task Group. J Occup Med. 1991;33:1221–9. 1800677

[6] Altpeter E, Burnand Bernard, Capkun G, Carrel R, Cerutti B, Mäusezahl-Feuz M, Gassner M, Junker C, Künzli N, Lengeler C, Minder C, Rickenbach M, Schorr D, Vader J-P, Zemp E (2005). Essentials of Good Epidemiological Practice. Soz Präventivmed.

[7] Olsen J. Good Epidemiological Practice (GEP). Proper conduct in epidemiologic research. Prepared for the IEA Brazil meeting April, 2007. Retrieved from http://alraziuni.edu.ye/book1/Health%20and%20Society/Good%20Epidemiological%20Practice%20(GEP)%20Proper%20Conduct%20in%20Epidemiologic%20Research.pdf. Accessed 29 Oct 2018.

[8] Swaen GMH, Langendam M, Weyler J, Burger H, Siesling S, Atsma WJ, Bouter L (2018). Responsible epidemiologic research practice: a guideline developed by a working group of the Netherlands Epidemiological Society. J Clin Epidemiol.

[9] Preliminary GEP guidelines. Appendix to the document “Good Epidemiological Practice—compendium of best practices and guidelines for research conducted at KIT”. KIT Health Royal Tropical Institute, Amsterdam. https://213ou636sh0ptphd141fqei1-wpengine.netdna-ssl.com/health/wp-content/uploads/sites/4/2017/06/GEP-Compendium-Annex-1-KIT-guidelines-Preliminary_v03.05.2018.pdf (2018). Accessed 19 Oct 2918.

[10] GPS—good practice in secondary data analysis: revision after fundamental reworking, 3rd edition 2012, slightly modified in 2014. German Society for Epidemiology (DGEpi). Retrieved from https://www.dgepi.de/assets/Leitlinien-und-Empfehlungen/1a6f57cb65/GPS_revision2-final_august2014.pdf (2014). Accessed 23 Oct 2018.

[11] Augustin J, Kistemann T, Koller D, Lentz S, Maier W, Moser J, Schweikart J. Good cartographical practice in the healthcare system (GKPiG). https://www.ssoar.info/ssoar/handle/document/52071. Social Science Access Repository. April 2017. Accessed 5 Oct 2018.

[12] Starke D, Tempel G, Butler J, Starker A, Zühlke C, Borrmann B. Gute Praxis Gesundheitsberichterstattung—Leitlinien und Empfehlungen. [Good practice in health reporting—guidelines and recommendations]. Robert Koch Institute. 10.17886/rki-gbe-2017-001 (2017). Accessed 23 Oct 2018.

[13] Retrieved from the methods and infrastructure for networked medical research (TMF). http://www.tmf-ev.de/EnglishSite/Home. Accessed 23 Oct 2018.

[14] Enhancing the quality and transparency of health research. Retrieved from http://www.equator-network.org. Accessed 22 Oct 2018.

[15] German Evaluation Society DeGEval. [Standards für evaluation]. Retrieved from http://www.degeval.de/degeval-standards/standards-fuer-evaluation/. Accessed 18 Oct 2018.

[16] The future of research communications and e-scholarship (FORCE11). FAIR-principles. Retrieved from https://www.force11.org/fairprinciples. Accessed 18 Oct 2018.

[17] Open access to scientific information. Retrieved from https://open-access.net/DE-EN/germany-english/. Accessed 18 Oct 2018.

[18] Schwerpunkt initiative “digitale information”. Retrieved from https://www.allianzinitiative.de/about-us/?lang=en. Accessed 23 Oct 2018.

[19] DFG guidelines on the handling of research data. Deutsche Forschungsgemeinsachaft. http://www.dfg.de/download/pdf/foerderung/antragstellung/forschungsdaten/guidelines_research_data.pdf (2015). Accessed 23 Oct 2018.

[20] Leitlinien und Empfehlungen zur Sicherung von Guter Epidemiologischer Praxis (GEP). [Guidelines and ecommendations for ensuring Good Epidemiological Practice (GEP)] https://www.dgepi.de/assets/Leitlinien-und-Empfehlungen/66777155c7/Leitlinien_fuer_Gute_Epidemiologische_Praxis_GEP_vom_September_2018.pdf. German Society for Epidemiology (DGEpi) (2018). Accessed 23 Oct 2018.

[21] World Medical Association. WMA declaration of Helsinki—ethical principles for medical research involving human subjects. Most recently amended by the 64th WMA General Assembly. Fortaleza (Brazil). https://www.wma.net/policies-post/wma-declaration-of-helsinki-ethical-principles-for-medical-research-involving-human-subjects/ (2013). Accessed 18 Oct 2018.

[22] Council of Europe. Convention for the protection of human rights and dignity of the human being with regard to the application of biology and medicine: convention on human rights and biomedicine (bioethics convention). ETS No.164. Oviedo. https://rm.coe.int/168007cf98 (1997). Accessed 18 Oct 2018.

[23] Harnischmacher U, Ihle P, Berger B, Goebel JW, Scheller J (2006). Checkliste und Leitfaden zur Patienteneinwilligung—Grundlagen und Anleitung für die klinische Forschung [Patient consent checklist and guide—the basics and guidance for clinical research].

[24] Hansen MM, Miron-Shatz T, Lau AYS, Paton C (2014). Big data in science and healthcare: a review of recent literature and perspectives: contribution of the IMIA Social Media Working Group. Yearb Med Inform.

[25] Binder H, Blettner M (2015). Big data in medical science—a biostatistical view. Part 21 of a series on evaluation of scientific publications. Dtsch Arztebl Int.

[26] Lipworth W, Mason PH, Kerridge I, Ioannidis JPA (2017). Ethics and epistemology in big data research. J Bioeth Inquiry.

[27] Kaplan RM, Chambers DA, Glasgow RE (2014). Big data and large sample size: a cautionary note on the potential for bias. Clin Transl Sci.

[28] STRATOS (Strengthening analytical thinking for observational studies) initiative. Retrieved from http://www.stratos-initiative.org. Accessed 18 Oct 18.

[29] Sauerbrei W, Abrahamowicz M, Altman DG, le Cessie S, Carpenter J, on behalf of the STRATOS initiative (2014). STRengthening analytical thinking for observational studies: the STRATOS initiative. Stat Med.

[30] Röhrig B, du Prel J-B, Wachtlin D, Blettner M (2009). Studiendesign in der medizinischen Forschung. Teil 2 der Serie zur Bewertung wissenschaftlicher Publikationen [Study design in medical research. Part 2 of the series for the evaluation of scientific publications]. Dtsch Arztebl Int.

[31] Röhrig B, du Prel J-B, Wachtlin D, Blettner M (2009). Studiendesign in der medizinischen Forschung. Teil 3 der Serie zur Bewertung wissenschaftlicher Publikationen [Study design in medical research. Part 3 of the series for the evaluation of scientific publications]. Dtsch Arztebl Int.

[32] Standards für Evaluation [standards for evaluation]. DeGEval—gesellschaft für evaluation. 2016. Retrieved from http://www.degeval.de/degeval-standards/standards-fuer-evaluation/. Accessed 18 Oct 2018.

[33] Rienhoff O, Semler SC. Terminologien und Ordnungssysteme in der Medizin—Standortbestimmung und Handlungsbedarf in den deutschsprachigen Ländern. Standortbestimmung und Handlungsbedarf in den deutschsprachigen Ländern [Terminology and classification systems in medicine—determination of position and need for action in the German-speaking countries]. 1st ed. Berlin: MWV Medizinisch Wissenschaftliche Verlagsgesellschaft; 2015.

[34] DAE, GMDS, DGSMP. Messung und Quantifizierung soziographischer Merkmale in epidemiologischen Studien [Measurement and quantification of sociographic features in epidemiological studies]. Retrieved from German Society for Epidemiology. https://www.dgepi.de/assets/Leitlinien-und-Empfehlungen/708cf24a3d/Messung-und-Quantifizierung-soziodemographischer-Merkmale.pdf (1997). Accessed 18 Oct 2018.

[35] Michalik C, Ngounongo S, Stausberg J. Von der Evaluierung zur Konsolidierung: Anforderungen an Kohortenstudien & Register IT—KoRegIT [From evaluation to consolidation: requirements for cohort studies & register IT—KoRegIT]. Berlin; 2015.

[36] Deutsche Arbeitsgemeinschaft für Epidemiologie (DAE). Epidemiologie und Datenschutz. [Epidemiology and data protection]. June 1998. Retrieved from Germany Society for Epidemiology. https://www.dgepi.de/assets/Leitlinien-und-Empfehlungen/02f5f11feb/Epidemiologie-und-Datenschutz.pdf. Accessed 18 Oct 2018.

[37] Pommerening K, Drepper J, Helbing K, Ganslandt T (2014). Leitfaden zum Datenschutz in medizinischen Forschungsprojekten—Generische Lösungen der TMF 2.0” [Guideline for data protection in medical research projects. TMF’s generic solutions 2.0.].

[38] EU General Data Protection Regulation 2016/679. Article 4. Definitions. Retrieved from http://www.privacy-regulation.eu/en/article-4-definitions-GDPR.htm. Accessed 18 Oct 2018.

[39] Kiehntopf M, Böer K (2008). Biomaterialbanken—Checkliste zur Qualitätssicherung. [Biomaterial banks—checklists for quality assurance] under collaboration of J. Goebel.

[40] Stellungnahme der Deutschen Gesellschaft für Humangenetik zu genetischen Zusatzbefunden in Diagnostik und Forschung [Statement of the German Society for Human Genetics on additional genetic findings in diagnostics and research]. Deutsche Gesellschaft für Humangenetik e.V. May 2013. https://www.gfhev.de/de/leitlinien/LL_und_Stellungnahmen/2013_05_28_Stellungnahme_zu_genetischen_Zusatzbefunden.pdf. Accessed 18 Oct 2018.

[41] Nonnemacher M, Nasseh D, Stausberg J. Datenqualität in der medizinischen Forschung—Leitlinie zum adaptiven Management von Datenqualität in Kohortenstudien und Registern [Data quality in medical research—Guideline for adaptive data quality management in cohort studies and registries]. 2nd updated and revised ed. Berlin: MWV Medizinisch Wissenschaftliche Verlagsgesellschaft; 2014.

[42] Schönbrodt FD, Gollwitzer M, Abele-Brehm AE (2017). Der Umgang mit Forschungsdaten im Fach Psychologie: Konkretisierung der DFG-Leitlinien [The handling of research data in the field of psychology: concretization of the DFG guidelines]. Im Auftrag des DGPs Vorstands (17.09.2016). Psychol Rundsch.

[43] Empfehlungen zur Spezifikation des Kerndatensatz Forschung (Drs. 5066-16). Wissenschaftsrat. http://www.wissenschaftsrat.de/download/archiv/5066-16.pdf (2016). Accessed 22 Oct 2018.

[44] Huebner M, Vach W, le Cessie S (2016). A systematic approach to initial data analysis is good research practice. J Thorac Cardiovasc Surg.

[45] Allianz der Wissenschaftsorganisation. Grundsätze zum Umgang mit Forschungsdaten. https://www.wissenschaftsrat.de/download/archiv/Allianz_Grundsaetze_Forschungsdaten.pdf (2010). Accessed 22 Oct 2018.

[46] Data Citation Synthesis Group: Joint Declaration of Data Citation Principles. Martone M. (ed.) San Diego CA. FORCE11. 10.25490/a97f-egyk (2014). Accessed 22 Oct 2018.

[47] Schneider UK (2015). Sekundärnutzung klinischer Daten—Rechtliche Rahmenbedingungen mit einem Beitrag von Alexander Roßnagel und Gerrit Hornung.

[48] Moher D, Altman D, Schulz K, Simera I, Wager E (2014). Guidelines for reporting health research: a user’s manual.

[49] von Elm E, Altman DG, Egger M, Pocock SJ, Gøtzsche PC, Vandenbroucke JP, STROBE Initiative (2007). The strengthening the reporting of observational studies in epidemiology (STROBE) statement: guidelines for reporting observational studies. PLoS Med.

[50] Benchimol EI, Smeeth L, Guttmann A, Harron K, Moher D, Petersen I, Sørensen HT, von Elm E, Langan SM, RECORD Working Committee (2015). The reporting of studies conducted using observational routinely-collected health data (RECORD) statement. PLoS Med.

[51] Moher D, Liberati A, Tetzlaff J, Altman DG, The PRISMA Group (2009). Preferred reporting items for systematic reviews and meta-analyses: the PRISMA statement. PLoS Med.

[52] Enhancing the quality and transparency of health research. Retrieved from http://www.equator-network.org. Accessed 22 Oct 2018.

[53] Von Elm E, Altman DG, Egger M, Pocock SJ, Gøtzsche PC, Vandenbroucke JP (2007). Strengthening the reporting of observational studies in epidemiology (STROBE) statement: guidelines for reporting observational studies. BMJ.

[54] Gallo V, Egger M, McCormack V, Farmer PB, Ioannidis JP, Kirsch-Volders M, Matullo G, Phillips DH, Schoket B, Stromberg U, Vermeulen R, Wild C, Porta M, Vineis P (2011). Strengthening the reporting of observational studies in epidemiology-molecular epidemiology (STROBE-ME): an extension of the STROBE statement. Eur J Epidemiol.

[55] Swart E, Bitzer EM, Gothe H, Harling M, Hoffmann F, Horenkamp-Sonntag D, Maier B, March S, Petzold T, Röhrig R, Rommel A, Schink T, Wagner C, Wobbe S, Schmitt J (2016). A consensus german reporting standard for secondary data analyses, Version 2. (STROSA-standardisierte berichtsroutine für sekundärdatenanalysen). Gesundheitswesen.

[56] Buttmann-Schweiger N, Grube M, Zeeb H für die Initiativgruppe „besser forschen“der DGEpi. Open access survey der DGEpi, DGSMP und der IBS-DR. Handreichung Open Access und Repositorien. 2017; 2018, 10.5281/zenodo.250527. Accessed 23 October.

[57] Lesch W, Schütt A (2016). Gesundheitsforschung kommunizieren, Stakeholder Engagement gestalten. Grundlagen, Praxistipps und Trends.

[58] Guidance Manual and Rules for Guideline Development. Network of Scientific Medical Societies in Germany AWMF. https://www.awmf.org/fileadmin/user_upload/Leitlinien/AWMF-Regelwerk/AWMF-Guidance_2013.pdf (2012). Accessed 23 Oct 2018.

